# Isolated complete caudate lobectomy with Glissonean pedicle isolation using Takasaki’s technique and right–left approach: preliminary experience from two case reports

**DOI:** 10.1186/s12957-022-02496-3

**Published:** 2022-02-04

**Authors:** Ham Hoi Nguyen, Thanh Khiem Nguyen, Van Duy Le, Tuan Hiep Luong, Kim Khue Dang, Vu Quang Nguyen, Hong Son Trinh

**Affiliations:** 1 Department of Gastrointestinal and Hepato-pancreato-biliary Surgery, Bach Mai Hospital, Hanoi, Vietnam; 2 Department of Surgery, Hanoi Medical University, 1st Ton That Tung Street, Dong Da, Hanoi, 11521 Vietnam; 3 Department of Oncology, Viet Duc University Hospital, Hanoi, Vietnam

**Keywords:** Caudate lobe, Glissonean pedicle, Right, left-side approach, Liver tumor

## Abstract

**Background:**

Tumors located in the caudate lobe may be primary tumor or metastases from other sites. Isolated caudate lobectomy (ICL) is a challenging procedure due to its complex structure and location. The access route to the caudate lobe has an important role in the success of the operation.

**Methods:**

Based on the characteristics of the segment I location, which is the part of the liver located in front of the vena cava, below the hepatic veins, and cranial to the hilar plate, our approach aims to isolate the entire caudate lobe from these anatomical structures with the following steps: dissecting the caudate lobe from the hilar plate and isolating the caudate lobe from the IVC and from the hepatic veins along with parenchymal resection.

**Results:**

We report two successful cases with the Glissonean pedicle transection method described by Takasaki and the combined right- and left-side approach: a 63-year-old female patient with a 46-mm-in-diameter HCC tumor and a 39-year-old female patient with a 45-mm lesion and the pathological result was focal nodular hyperplasia.

**Conclusions:**

We found this to be a safe and effective approach, which can be applied to all cases of benign tumors or in the case of malignant tumors located entirely in the caudate lobe when extended hepatic resection is not possible due to poor liver function or small remnant liver volume.

## Background

The caudate lobe is the part of the liver that lies behind the Glissonean pedicle (GP) and three hepatic veins (HVs), before the inferior vena cava (IVC). According to Kumon classification, the caudate lobe (CL) is divided into three regions: the Spigel lobe, the process portion or the caudate process, and the paracaval portion [1]. Tumors of CL may be a primary tumor in the liver or a tumor from another site that has metastasized. Because of its anatomical location that is strongly related to the GP, the HV anteriorly, and the short HV passes directly from the parenchyma of the CL to the vena cava and is posteriorly related to the anterior aspect of the vena cava, therefore, complete caudate lobectomy (CCL) (an anatomic resection of segment I and segment IX resection with Couinaud’s classification [2]) is a huge challenge for the surgeons. Isolated CCL can be performed alone or in combination with right lobectomy and right and left hepatectomy. Isolated CCL is indicated for benign tumors located in all parts of this liver region [3]. For hepatic malignancy, especially in patients with hepatocellular carcinoma (HCC), often associated with cirrhosis, individual caudate lobectomy is proposed for tumors localized in this region to preserve remaining liver function.

Generally, the approach of CL has four ways including right, left side, right and left coordination, and anteriorly through the parenchymal cut of the liver. Depending on the location, characteristics of the tumor, and the type of caudate lobectomy, the surgeon will have a suitable approach to the tumor [4–7]. For complete caudate lobe resection, we found that the right and left two-way approach combined with GP control by Takasaki’s technique is an easy and safe method. Herein, we report two cases of a successful GP isolation using Takasaki’s technique and the right–left approach. All our work has been reported in line with the SCARE criteria and guidelines [8].

## Methods: surgical procedure

**Fig. 1:**
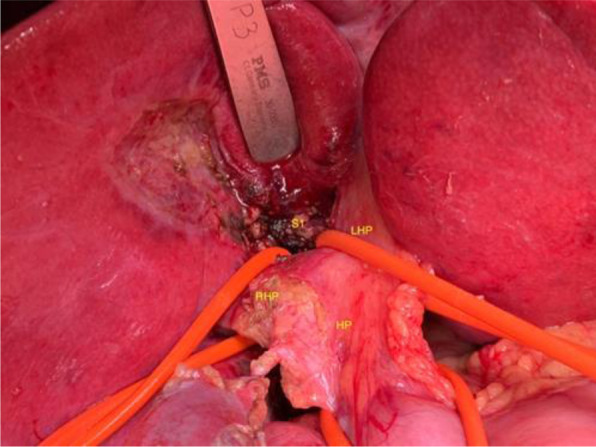
Right and left hepatic pedicles after controlling with tapes using Takasaki’s technique. S1 segment I, LHP left hepatic pedicle, RHP right hepatic pedicle, HP hepatic pedicle

**Fig. 2:**
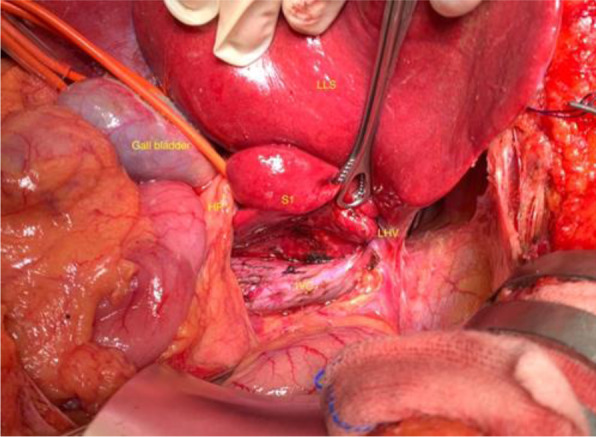
Ligation of the short hepatic veins from the subsegment I to the inferior vena cava. S1 segment I, LLS left lateral section, LHV left hepatic vein, IVC inferior vena cava, HP hepatic pedicle

**Fig. 3:**
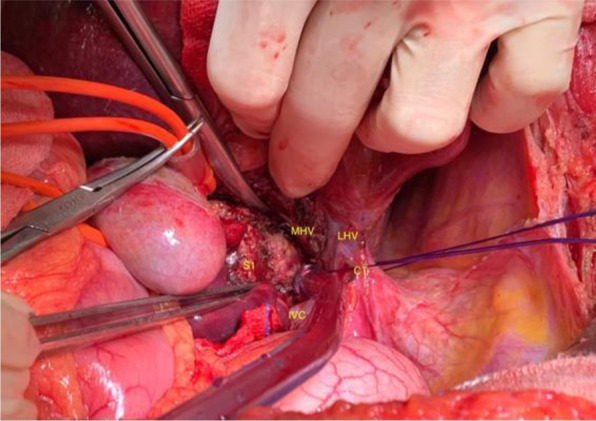
Parenchymal resection. Usually starting from the left side, the liver parenchyma is resected just below the Arantius ligament, separating the liver parenchyma from the inferior surface of the middle and left hepatic veins. At the bottom, lifting the right hepatic pedicle by using tapes, cut the caudate process and separate it from the right liver parenchyma. S1 segment I, IVC inferior vena cava, CT common trunk, LHV left hepatic vein, MHV medial hepatic vein

**Fig. 4:**
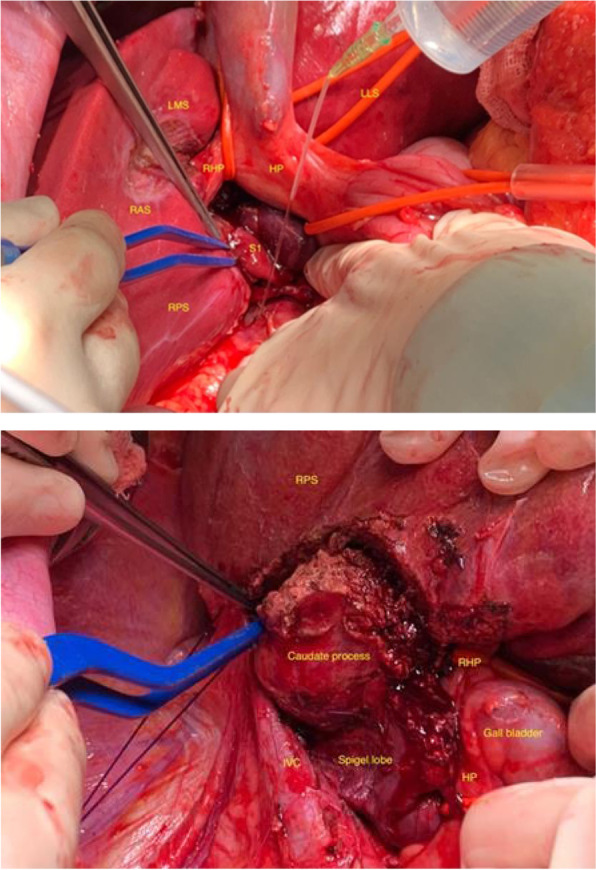
Parenchymal resection. The liver parenchymal cutline from the right posterior approach after cutting the liver parenchyma from the left to the right border of the middle hepatic vein, the boundary is determined after ligation of the right hepatic pedicle of subsegment 1. S1 segment I, LLS left lateral section, LMS left medial section, RAS right anterior section, RPS right posterior section, RHP right hepatic pedicle, HP hepatic pedicle

**Fig. 5:**
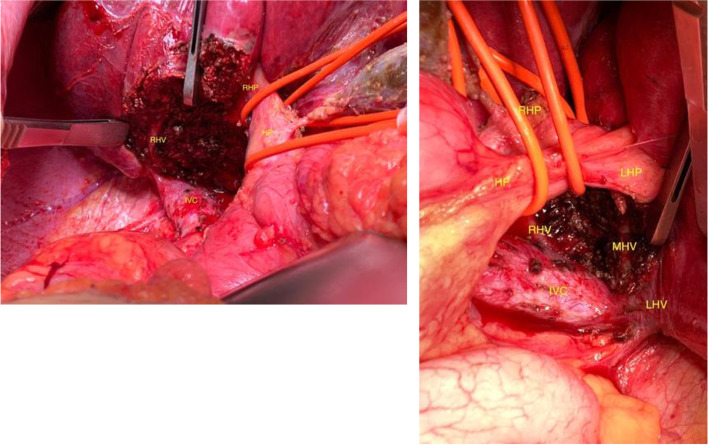
Resected liver parenchyma. LHV left hepatic vein, MHV medial hepatic vein, RHV right hepatic vein, RHP right hepatic pedicle, HP hepatic pedicle, IVC inferior vena cava

Based on the characteristics of the segment I location, which is the part of the liver located in front of the vena cava, below the HVs, and behind the GP, our approach aims to separate the entire CL from these anatomical structures with three main steps:

- *Step 1: Separation of the caudate lobe from the hepatic pedicle* by using Takasaki’s approach to the pedicle including cholecystectomy, dissection of the *cystic plate and umbilical plate* (thickened parts of the GP comprising collagen-rich connective, which was first described by Couinaud [9]), and resection of *the anchors* (the thin, cord-like structures located at the orifices of the GP that connect with Laennec’s capsule [9]); isolate the right and left hepatic pedicles and maximally move these pedicles (Fig. 1 in the Appendix). Dissect clearly the right side of the segment I pedicle coming from the posterior right hepatic pedicle (usually about 1cm from the posterior segmental pedicle). With the left-side approach, often after opening of the lesser omentum, segment I is pushed posteriorly to the Arantius fascia to show its pedicle. Usually, there are two pedicles entering segment I from the left hepatic pedicle.
- *Step 2: Separation of the caudate lobe from the IVC*: cut the coronary and triangular ligaments to release the right and left lobes and ligate all the veins of the segment I directly fall into the anterior aspect of the IVC in two successive directions (Fig. 2 in the Appendix): right and left sides. From the left, lift the Spigel lobe and ligate the entire vein into this part up to the LHV. Tie the vein into the caudate process. From the right, ligate the inferior hepatic veins (accessory RHVs) and cut the hepatocaval ligament, until the right and left sides are connected.
- *Step 3: Separation of the caudate lobe from the hepatic veins*, which is equivalent to parenchymal resection: using both the left and right approach and an upward path to cut the caudate process. *Usually starting from the left side*, the liver parenchyma is resected just below the Arantius ligament, separating the liver parenchyma from the inferior surface of the middle and left HVs. At the bottom, lifting the right hepatic pedicle by shaking, cut the caudate process and separate it from the right liver parenchyma; this cut will meet the section cut from the left side; an inferior approach will resolve parenchymal resection of the lower right quadrant of segment I (Fig. 3 in the Appendix). *Continue moving to the right approach*: the parenchymal opening in this side will loop up from the RHV root and down to meet the caudal process section made from below (Fig. 4 in the Appendix). It is important to note that this parenchymal opening should be directed toward parenchymal resection adjacent to the inferior surface of the RHV. When dissection found the landmark of the RHV, continue to cut parenchyma until the left side cut and remove the CL. During parenchymal resection, there are selective right and left pedicle clamps when approached from each side, respectively.
- Hemostasis of liver resected parenchyma (Fig. 5 in the Appendix). Place a cystic duct catheter to check for bile leaks.

## Results

The above technique was successfully performed on 2 patients.

**Fig. 6:**
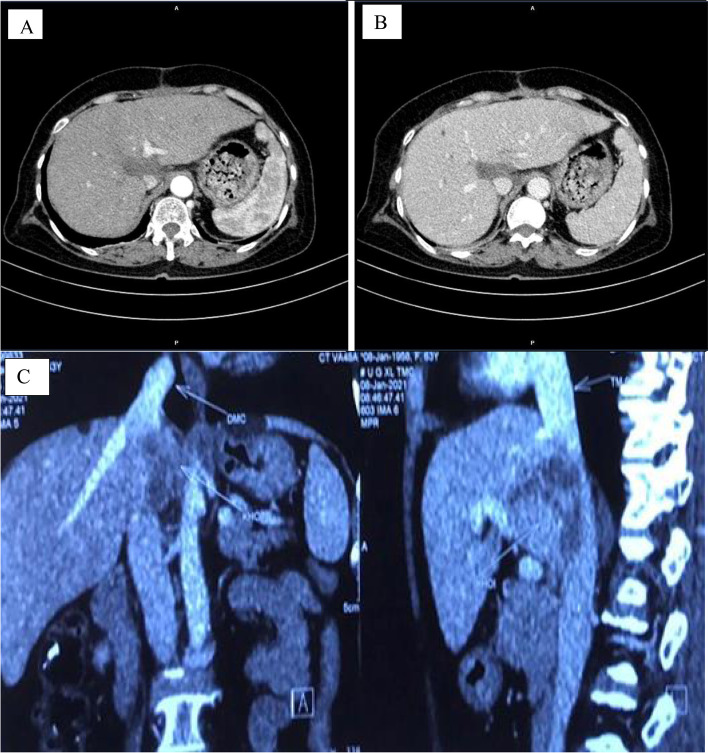
The CT result of the first patient showed a tumor occupying nearly the entire subsegment I with characteristics of HCC (**A**, **B**), at size 46 mm, compressing inferior vena cava, close to the hepatic pedicle, right and left hepatic veins but not invasive, no thrombus portal vein (**C**)

The first case was a 63-year-old female patient with a history of hepatitis B without treatment for many years, and a liver tumor was discovered because of abdominal pain. Computed tomography (CT) result showed a tumor occupying nearly the entire subsegment I with characteristics of HCC, at size 46 mm, compressing the inferior vena cava, close to the hepatic pedicle, right and left hepatic veins but not invasive, no thrombus portal vein (Fig. 6 in the Appendix), and serum AFP > 400 ng/ml. Liver function tests are normal. During the resection of the liver parenchyma, we injured the middle hepatic vein. We managed by lifting the tapes over the common trunk of the left and middle hepatic veins, with a temporary Pringle maneuver technique and suture of the lesion with Prolen 5/0. The operation time was 130 min, and the amount of blood lost during the surgery was about 250ml. Injection of an isotonic solution of sodium chloride through the cystic duct via a catheter did not show any bile leakage. Postoperatively stable, without complications, the patient was discharged after 7 days. The pathology is that the hepatocellular carcinoma moderately differentiated occupies nearly the entire subsegment I, the resection margin is less than 0.5 cm, and there was no cancer. In the follow-up 9 months after surgery, there is no recurrence on CT and magnetic resonance imaging (MRI). The first patient was presented with cirrhosis. However, she had no relevant portal hypertension, due to normal platelet counts, and no sign of varices on upper and lower endoscopy. So, she had a preserved liver function before surgery.

**Fig. 7:**
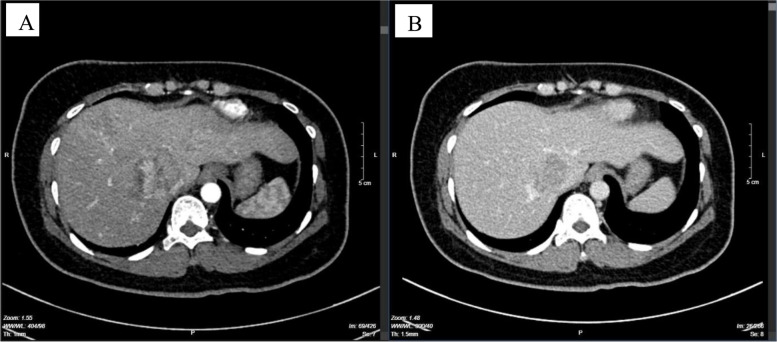
The CT result of the second patient showed a 45-mm-size tumor of subsegment I, decreased density before injection, rapid drug enhancement in the arterial phase (**A**), rapid drug removal in venous phase, and irregular margins (**B**)

**Fig. 8:**
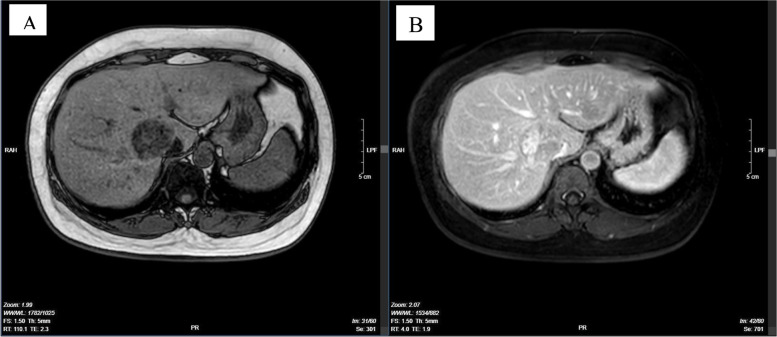
Liver MRI of the second patient showed a liver tumor about 5cm in size, decreased signal on T1W out phase (**A**), rapid enhancement in the arterial phase and rapid drug elimination in the venous phase, clearly limited but close to the hepatic veins and inferior vena cava (**B**)

The second case was a 39-year-old female patient with no medical history, routine examination to detect liver tumor, serum AFP level was 3.03 ng/ml; HbsAg and HCV results were negative. CT result showed a 45-mm-size tumor of subsegment I, decreased density before injection, early enhancement in arterial phase, rapid washout in portal venous phase, and irregular margins (Fig. 7 in the Appendix). No portal vein thrombosis and no invasion of the hepatic pedicle, vena cava, or hepatic veins were found. There were no peritoneal fluid and no abnormal lymph nodes. Liver MRI showed a liver tumor about 5cm in size, decreased signal on T1W out phase and increased signal on T2W, diffusion, early enhancement in arterial phase, rapid washout in portal venous phase, and clearly limited but close to the hepatic veins and inferior vena cava (Fig. 8 in the Appendix). Gastroscopy and colonoscopy were normal. Imaging features did not allow excluding hepatocellular carcinoma, and tumor biopsies are difficult due to tumor location, so we decided to remove the entire caudate lobe according to the above technique. Surgery time was 120 min, there were no complications during surgery, and blood loss during surgery was about 150ml. Injection of an isotonic solution of sodium chloride through the cystic duct via a catheter did not show any bile leakage. After surgery, the patient was stable, without complications, and was discharged after 7 days of hospital stay. Postoperative pathology is focal nodular hyperplasia, and resection margin is less than 0.5 cm, not involved. After 7 months of follow-up, the patient is now healthy and has normal daily activities.

## Discussion

**Fig. 9:**
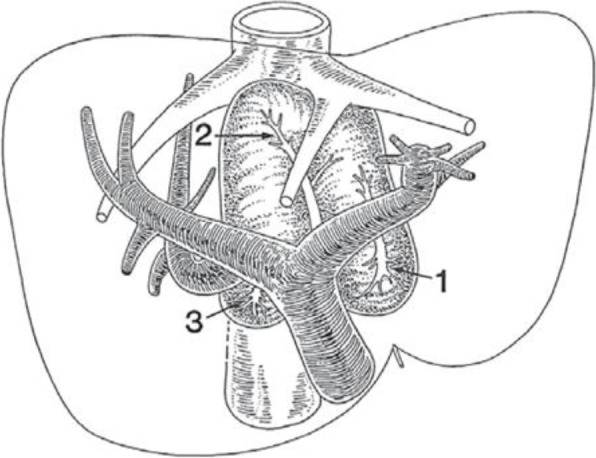
The caudate lobe, or subsegment I according to Kumon’s classification [1], is the portion of the liver that lies between the vascular structures: the portal vein posteriorly, the hepatic pedicle below, and the hepatic veins superiorly

The caudate lobe, or subsegment I according to Kumon’s classification, is the portion of the liver that lies between the vascular structures: the portal vein posteriorly, the hepatic pedicle below, and the hepatic veins superiorly. Kumon divides the subsegment I into 3 parts: the Spigel lobe, also known as S1l, which lies below the lesser omentum and extends to the left posterior hepatic part of the vena cava; the anterior venous part is the part located in front of the vena cava and to the right of the Spigel lobe, also known as S1r, which is closely associated with the right hepatic vein and the middle hepatic vein; and the caudal process is the right portion of the anterior venous part also known as S1c [1] (Fig. 9 in the Appendix).

**Fig. 10:**
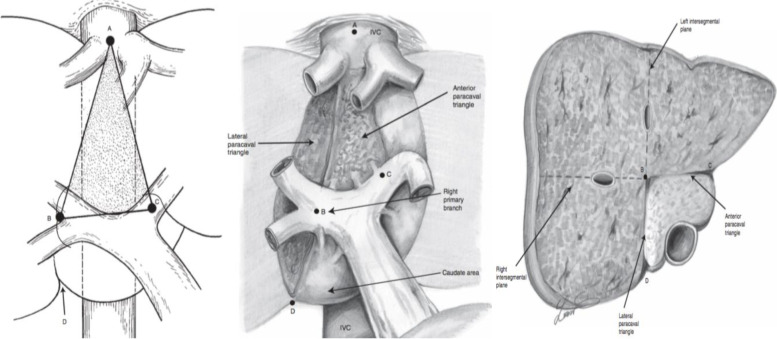
The anterior triangle of the vena cava separates the left and caudal lobes, and the lateral triangle of the vena cava separates the caudal lobe from the right lobe [21]

According to Takasaki, the liver has 4 main regions: the right segment, the middle segment, the left segment, and the caudal region. The caudal region occupies about 10% of liver volume and is supplied by several small Glisson pedicles directly from the first branch at the hepatic hilum. This is the region of the liver located in front of the vena cava, whose veins drain directly into the vena cava. However, this part of the liver is only adjacent but not attached to the vena cava. The boundaries of the three lobes of the liver with the caudal region are delimited by triangles as shown in the figure. The anterior triangle of the vena cava separates the left and caudal lobes, and the lateral triangle of the vena cava separates the caudal lobe from the right lobe [10] (Fig. 10 in the Appendix).

The above two anatomical perspectives are the basis for our approach to finding and ligation of the subsegment I’s vessels from the right hepatic pedicle or left hepatic pedicle. The detachment of the hilum plate, the right and left hepatic pedicle, and the ligation of the branches into the subsegment I also help separate this liver part from the pedicle to allow more space for dissection in this hard-to-reached area. The lateral and anterior triangle segment of the vena cava in Fig. 10 in the Appendix shows us the left and right liver resections to separate the subsegment I from the right and left liver lobes. From the left, an anatomical landmark that can be relied on is the Arantius’ ligament; the liver parenchymal resection must be close to this ligament to avoid injury to the middle and left hepatic vein. Kumon’s classification allows a 3-way parenchymal resection approach according to our technique from the left side (Spigel lobe), from below (the caudal process), and from the right side (the paracaval portion).

For a successful ICCL, in addition to understanding the careful anatomy in surgery, the choice of approach to liver tumor also plays a very important role. Depending on the location and pathology of tumor and the liver parenchyma’s quality, the appropriate approach and method should be selected to ensure radical oncology and to limit complications, especially liver failure after large tumor volume resection. Regarding the location for a rational approach, Hasegawa et al. [11] classified liver tumors originating from the caudate lobe into 5 types:

- Type 1: Lesions in Spigel lobe’s upper part
- Type 2: Lesions in Spigel lobe’s lower part
- Type 3: Lesions around the vena cava (paracaval portion)
- Type 4: Lesions in the caudate process
- Type 5: Diffuse lesions of the entire caudate lobe

Our first patient is a case of HCC, a tumor of type 5 according to Hasegawa’s classification; although the tumor laid closely in the right hepatic vein, the caudate and right lobectomy could be more radical, because of cirrhosis and the remaining liver volume was not enough. In the second case, the tumor completely occupied the anterior vena cava of the subsegment I and was pushed to the Spigel lobe and could be classified between types 3 and 5; after removal of the entire tumor for an immediate biopsy, the tumor was a benign lesion. Thus, our choice of total subsegment I resection in the two cases is reasonable in different circumstances.

During complete solitary caudate lobectomy, there are four main approaches: left-sided approach, right-sided approach, anterior approach, and right and left combined approach. For tumors in the left region (Spigel lobe), the left approach is sufficient, but for large tumors or tumors in the anterior area of vena cava, it is often necessary to combine with the right approach or use the anterior parenchymal opening [12]. The anterior parenchymal opening provides good visibility and access for subsegment I resection, especially for large tumors and closely related to the hepatic veins [13, 14], but it increases the risk of bleeding and prolongs the surgical time [15, 16]. A combined left and right approach is recommended for most tumors of the caudate lobe [5, 17], especially for tumors larger than 4 cm in diameter, which are of primary origin from the paracaval portion or in the entire caudate lobe, or those that are thought to be malignancies, and require total caudate lobectomy to remove the tumor.

The exposure and ligation of the short hepatic veins are a difficult task in subsegment I hepatectomy [5]. Ligation and resection of the short hepatic veins release the entire subsegment I from the anterior area of the vena cava and facilitate the management of the hepatic veins and control of hemostasis during parenchymal resection. We found that the process of controlling the short hepatic veins was not too difficult after extensive mobilization of the right and left liver, when combining from both the right and left sides together with meticulous and careful dissection.

Exposure and control of the hepatic veins with tapes is a maneuver that should be performed before parenchymal resection because the caudate lobe parenchyma is in contact with the posterior surface of the hepatic veins, so the risk of injury to these veins is relatively high. The move to cut the Arantius’ ligament will help control the left and middle hepatic veins more easily, but we do not cut this ligament, but only separate it from the liver for the purpose of using this ligament as a landmark to start cutting the liver parenchyma from the left side.

Resection of liver parenchyma is the most difficult and successful-decision stage, and cutting the liver parenchyma ensures no tumor rupture and no damage to the hepatic veins, especially the middle hepatic vein. Because the hepatectomy area is located deep, close to the hepatic veins, it is easy to damage the hepatic veins, and when damaged, it is very difficult to control and manage. The authors have used many ways to safely and effectively cut the liver parenchyma such as using the hanging maneuver procedure [18] or using some kind of coloring agents to better define the hepatectomy boundary [4] or using anterior parenchymal resection to provide better visibility, but anterior parenchymal resection increases operative time and increases the risk of bleeding [15, 16]. To secure the parenchymal resection, we suggest the following tactics: firstly, after a three-pronged approach like above, detachment of the hilum plate and ligation of the pedicles of subsegment I help to save space to dissection as well as recognize parenchymal ischemic area; the second is clamping selective right and left hepatic pedicles respectively when cutting the liver tissue from both sides and only clamping the entire hepatic hilum if the bleeding is difficult to control; the third is to note the landmarks of parenchymal cut on both sides, on the left is close to the Arantius’ ligament and on the right is go from the origin following the inferior border of the right hepatic vein, to ensure the best cutting area and avoid damage to the hepatic veins. In field of laparoscopy, although we have not experience with laparoscopic isolated total caudate lobectomy yet, we though it is possible to apply this technique by laparoscopy or using the robotic platform, with several recently reported articles [19, 20].

Our criteria for this technique for patients before undergoing an operation for HCC are as follows: single tumor less than 5cm, Child A (5–6 points), and no relevant portal hypertension (normal platelet counts, no sign of varices on upper and lower endoscopy). Due to several authors, the tumor larger than 5cm especially in the paracaval area is challenging and we would consider the anterior transhepatic approach, which is described by Yamamoto et al. [10]. Eighty percent of our patient in our country is presented with chronic hepatitis B. For patients presented with a tumor on the caudate lobe, we have no AFP level limit, due to the difficulty in the technique of TACE for tumor on this region. However, in a patient with a high level of AFP, we would be more cautious to extrahepatic metastasis, and as long as we could exclude the extrahepatic metastasis, we still carry on the operation. In aspects of minimum resection margin size, our desired margin for a patient with HCC is 0.5cm. However, if the patient underwent isolated caudate lobectomy, a negative margin was acceptable.

## Conclusions

The key to success for hepatectomy is understanding the anatomy of the caudate lobe, choosing the suitable approach, and performing careful and meticulous dissection. Takasaki’s management of the hepatic pedicle and using a combined right- and left-side approach is safe and effective for non-invasive and vessel-related tumors (Figs. 1, 2, 3, 4, 5, 6, 7, 8, 9 and 10).

## Data Availability

Data is available upon reasonable request and with permission of Bach Mai Hospital.

## Abbreviations

CCL: Complete caudate lobectomy
CL: Caudate lobe
CT: Computed tomography
FNH: Focal nodular hyperplasia
GP: Glissonean pedicle
IVC: Inferior vena cava
LHV: Left hepatic vein
HCC: Hepatocellular carcinoma
HV: Hepatic vein
MRI: Magnetic resonance imaging
PV: Portal vein
RHV: Right hepatic vein
